# The Wobbler Mouse Model of Amyotrophic Lateral Sclerosis (ALS) Displays Hippocampal Hyperexcitability, and Reduced Number of Interneurons, but No Presynaptic Vesicle Release Impairments

**DOI:** 10.1371/journal.pone.0082767

**Published:** 2013-12-11

**Authors:** Karina D. Thielsen, Jakob M. Moser, Thomas Schmitt-John, Morten S. Jensen, Kimmo Jensen, Mai Marie Holm

**Affiliations:** 1 Department of Biomedicine, Aarhus University, Aarhus, Denmark; 2 Department of Molecular Biology and Genetics, Aarhus University, Aarhus, Denmark; Inserm, France

## Abstract

Amyotrophic lateral sclerosis (ALS) is the most common adult-onset motor neuron disease. It is a fatal degenerative disease, best recognized for its debilitating neuromuscular effects. ALS however also induces cognitive impairments in as many as 50% of affected individuals. Moreover, many ALS patients demonstrate cortical hyperexcitability, which has been shown to precede the onset of clinical symptoms. The wobbler mouse is a model of ALS, and like ALS patients the wobbler mouse displays cortical hyperexcitability. Here we investigated if the neocortical aberrations of the wobbler mouse also occur in the hippocampus. Consequently, we performed extracellular field excitatory postsynaptic potential recordings in the CA1 region of the hippocampus on acute brain slices from symptomatic (P45-P60) and presymptomatic (P17-P21) wobbler mice. Significant increased excitation of hippocampal synapses was revealed by leftward shifted input/output-curves in both symptomatic and presymptomatic wobbler mice, and substantiated by population spike occurrence analyses, demonstrating that the increased synaptic excitation precedes the onset of visible phenotypic symptoms in the mouse. Synaptic facilitation tested by paired-pulse facilitation and trains in wobbler and control mice showed no differences, suggesting the absence of presynaptic defects. Immunohistochemical staining revealed that symptomatic wobbler mice have a lower number of parvalbumin positive interneurons when compared to controls and presymptomatic mice. This study reveals that the wobbler mouse model of ALS exhibits hippocampal hyperexcitability. We suggest that the hyperexcitability could be caused by increased excitatory synaptic transmission and a concomitant reduced inhibition due to a decreased number of parvalbumin positive interneurons. Thus we substantiate that wobbler brain impairments are not confined to the motor cortex, but extend to the hippocampus. Importantly, we have revealed more details of the early pathophysiology in asymptomatic animals, and studies like the present may facilitate the development of novel treatment strategies for earlier intervention in ALS patients in the future.

## Introduction

Amyotrophic lateral sclerosis (ALS) is a progressive and fatal neurodegenerative disease [1]. In both ALS patients and in animal models of the disease, neurons of the spinal cord, brainstem and motor cortex are affected [2]. The injury and loss of motor neurons result in debilitating symptoms such as spasticity, muscle atrophy, generalized weakness, paralysis, and eventually in death from respiratory failure [3]. Consequently ALS is most recognized for its neuromuscular effect; however ALS has also been shown to induce cognitive impairments in as many as 50% of affected individuals, pointing towards higher order defects [4,5]. Additionally ALS patients demonstrate cortical hyperexcitability, and at least for patients carrying the superoxide dismutase 1 (SOD1) mutation, the increased excitability precedes the onset of clinical symptoms [6,7]. The cortical hyperexcitability in ALS patients is likely the result of a combination of increased excitation and decreased inhibition [8]. The inhibitory system may be impaired due to the loss of inhibitory interneurons [9], reduced concentration of extracellular GABA [10] or degeneration of inhibitory circuits [8]. Currently only one FDA-approved drug, Riluzole, is available for treatment of ALS patients [3,11], prompting detailed analysis of animal models with the aim of identifying novel targets for early interventions and treatment. 

The wobbler mouse is a model of ALS [12,13]. At approximately three weeks of age the first signs of the condition with muscle weakness in the forelimbs, and a characteristic wobbling gait, begin to develop in the homozygous wobbler mice (*wr/wr*) and progress till death [12,14]. The wobbler mouse carries a point mutation in the *Vps54* gene, which leads to an amino acid substitution of a highly conserved leucine to a glutamine (L967Q) in the C-terminal of the Vps54 protein [15]. The Vps54 protein is part of the evolutionarily conserved heterotetrameric GARP-complex [16]. The phenotype of the wobbler mouse is most likely the result of destabilization of the Vps54 protein, and consequently decreased levels of Vps54 protein and GARP-complex, yet the mechanisms are still unknown [17]. The GARP-complex is involved in retrograde transport from both early and late endosomes to the trans-Golgi network (TGN), and as such the wobbler mouse displays a connection between retrograde vesicle transport and neurodegeneration [18]. There is also growing evidence for this connection in ALS patients, as seen by the involvement of other endosomal trafficking factors, namely VAPB [19], alsin [20,21], and Figure 4 [22] in ALS [23]. 

Like ALS patients, the wobbler mouse exhibits cortical hyperexcitability; demonstrated in acute brain slices [24]. Interestingly, hyperexcitability has also been documented in the SOD1(G93A) mouse model of ALS. Here cultured spinal motor neurons revealed increased excitability [25] which could be decreased by riluzole at a therapeutic relevant concentration [26]. These findings are supported by electrophysiological analyses of cultured cortical SOD1(G93A) neurons [27], again pointing at defects in the brain of ALS individuals. A recent study documented that hyperexcitability can be observed already in embryonic motor neurons from SOD1(G93A) spinal cord [28].

Inhibitory interneurons have also been implicated in ALS with a reduction in parvalbumin positive interneurons seen in the motor cortex of ALS patients [9] and later reports showing the same trend [29]. Interestingly, decreased density of parvalbumin positive interneurons has likewise been proven in the motor cortex of the wobbler mouse [24]. Moreover, Meyer et al. documented a decreased density of hippocampal GABAergic interneurons in wobbler mice, which could be ameliorated by progesterone treatment [30]. At least four distinct types of interneurons in the CA1 area of the hippocampus express parvalbumin [31], and these parvalbumin positive cells make up a large fraction of the interneurons in the area [32]. Additionally, the loss of parvalbumin positive interneurons in the hippocampus has been suggested to be involved in diseases such as schizophrenia and epilepsy, most likely due to subsequent shifts in the delicate balance of the excitation/inhibition relationship [33].

The hippocampus, which is critically important in learning and memory and communicates with every part of the neocortex [34], has previously been implicated in ALS [35-38]. The ubiquitinated inclusions which are a hallmark of ALS have also been found in the hippocampus of ALS patients [35,38-40], as has inclusions of the DNA/RNA binding protein TDP-43 [36]. TDP-43 has been shown to be pathophysiologically linked to ALS [39,41], and TDP-43 upregulation and redistribution have also been found in the spinal cord of the wobbler mouse [42].

There are many similarities between the wobbler mouse and ALS patients [13]; both in relation to the visible symptoms, but also regarding symptoms on the cellular level. These include, but are not limited to, impaired retrograde transport [17], cortical hyperexcitability [24], mitochondrial dysfunctions [43], neurofilament aggregation [12], ubiquitin-positive protein aggregates [42], and abnormalities in the expression and localization of TDP43 [42]. The many similarities make the wobbler mouse an excellent model of ALS. Moreover, the wobbler mouse offers an important opportunity to study a well-defined presymptomatic phase of the disease, which is highly problematic in ALS patients, allowing us to identify early signs of dysregulations and identify novel targets for future research. 

Hyperexcitability can be caused by either one, or a combination, of three main factors. Firstly, enhanced glutamatergic synaptic transmission, as a result of increased synaptic excitation and/or enhanced glutamate release. Secondly, increased cellular excitability, e.g. as a result of a reduced action potential firing threshold. And thirdly, reduced functionality of the inhibitory system, due to structural and/or functional impairments. The aim of the present study was to investigate if cortical alterations are only present in the neocortex of the wobbler mouse [24,44] or extend to the hippocampus [30]. To take full advantage of this established ALS model, we implemented studies in symptomatic animals, but importantly, we also analyzed mice at the presymptomatic phase, to investigate if hippocampal dysregulations occur before the onset of phenotypic symptoms. We found increased hippocampal excitation and a reduced number of interneurons, indicating hippocampal hyperexcitability in the brain of the wobbler mouse model of ALS. 

## Materials and Methods

### Ethics statement

Experiments and housing were conducted in strict accordance with institutional, national, and EU guidelines for the care and use of laboratory animals. Procedures were approved and monitored by the Animal Welfare Officer at the Department of Biomedicine, Health, Aarhus University. Wobbler mice were bred on a (C57BL6/J) background, and kept with a cycle of 12:12 hours of light:darkness, with unrestricted access to food and water. Additionally, to achieve the best possible growth and weight gain of the wobbler mice, the wobbler mice and their healthy littermates had their diet enriched with peanut butter. 

### Genotyping

Offspring was genotyped, using tail biopsies around postnatal day (P) 14, before the execution of experiments, and again after the sacrifice and completion of the experiments in order to verify the genotype. Genotyping of wobbler mice was previously published [15]. Later another type of genotyping was developed by Simon Cuhlmann (in the laboratory of Thomas Schmitt-John, Aarhus University) and then also utilized by Diana et al. [45]. Here we used the following primer pairs to distinguish the wobbler allele from the wild type. For the wild type allele the primers were as follows: *Vps54 – f413*; 5’-gct tct ctg ttg aag cca ca-3’ and *Vps54 – wt –rev*; 5’-ccc aga tct cgg cca tat tta-3’ resulting in a band at about 415 base pairs. The wobbler allele was identified by the primer pair: *Vps54 – wr- f*; 5’-AGG CCT TAA AGA TCT GGA TCA-3’ and *Vps54 rev255*; 5’-tgc tcc tta ctc agg gat gc-3’ giving a band at about 260 base pairs. Annealing temperature was 63 °C. 

### Preparation of brain slices for electrophysiology

Wobbler mice (*Vps54* ^*wr/wr*^) and age-matched control littermates (*Vps54* ^*+/+*^ and *Vps54* ^*+/wr*^) of both sexes were anesthetized with isoflurane before decapitation. The brains were carefully dissected out and immediately transferred to ice-cold artificial cerebrospinal fluid (ACSF, in mM): 126 NaCl, 2.5 KCl, 1.25 NaH2PO4, 2.5 CaCl2, 1.3 MgCl2, 26 NaHCO3, 10 D-glucose, bubbled with carbogen (5% CO2-95% O2). 400 μm thick coronal slices containing the hippocampus were cut on a Vibratome 3000 (The Vibratome Company, Missouri, USA) and allowed to rest at least 1½ hours at room temperature in ACSF, before recordings were made.

### Electrophysiological recordings

**Figure 1:**
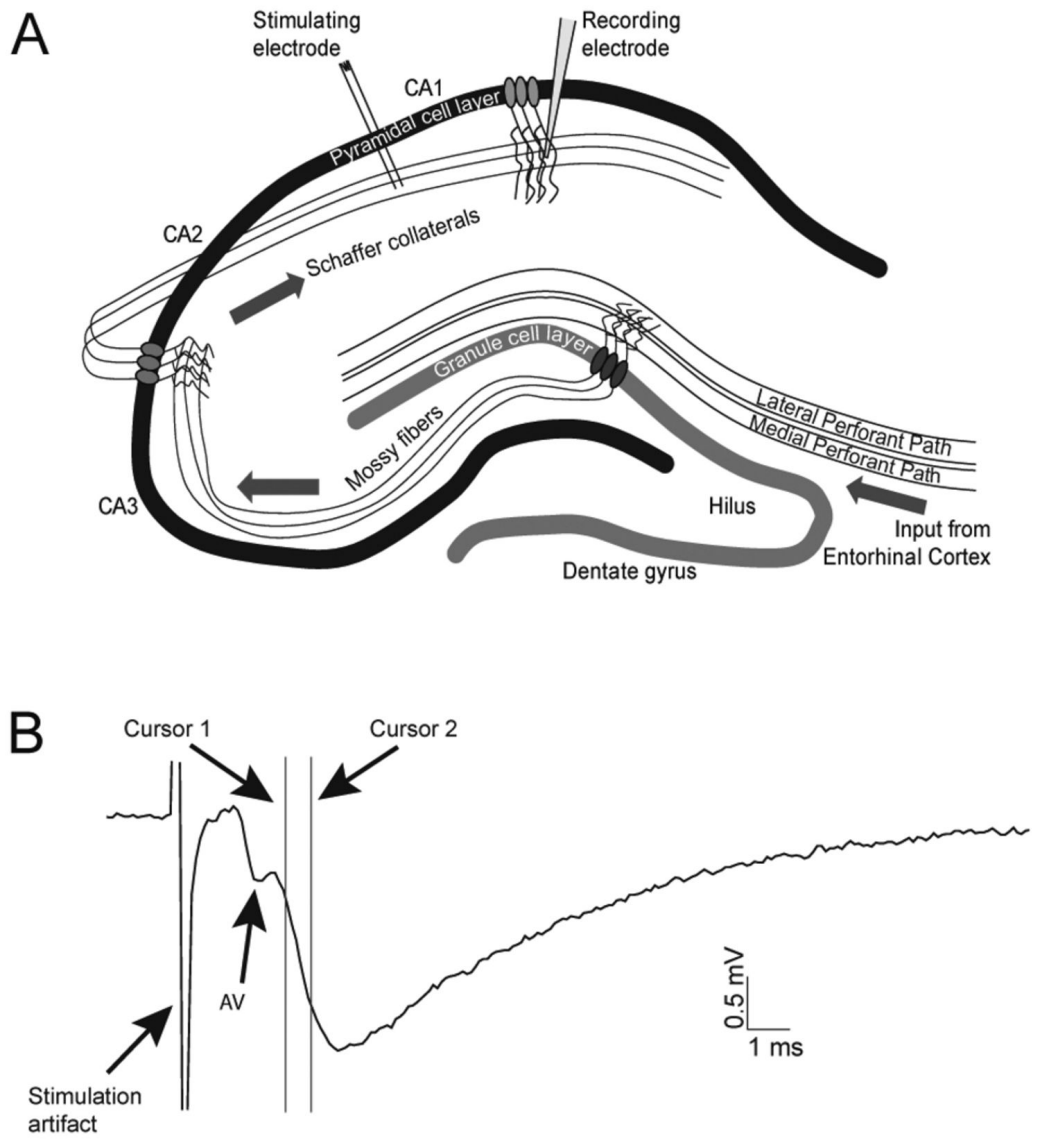
The hippocampal circuit and experimental setup. (A) Illustration of the tri-synaptic hippocampal circuit and placement of electrodes for our electrophysiological analyses. Input from the entorhinal cortex enters the dentate gyrus via the perforant pathway forming synapses on the dentate gyrus granule cells. The granule cells project axons, the mossy fibers, to the CA3 pyramidal neurons. The axons from the CA3 cells, the Schaffer collaterals, form synapses on the dendrites of the CA1 pyramidal cells [34]. Arrows indicate the direction of the neurotransmission. In all electrophysiological experiments described in this study the stimulating and recording electrodes were placed as pictured. (B) A representative trace obtained during a recording of the fEPSP. Cursors indicate the region of the rising phase of the fEPSP used to estimate the slope of the response. The slope is linearly related to the synaptic conductance and can be used as a measure of the activation of glutamatergic receptors in the postsynaptic membrane of Schaffer collateral synapses [49]. An arrow indicates the partially blanked stimulation artifact resulting from the brief electrical stimulation transient applied by the bipolar stimulation electrode. The afferent fiber volley (AV) is a result of the action potentials in the population of Schaffer collaterals traveling by the recording electrode and reflects the strength of the afferent input.

Extracellular field excitatory postsynaptic potentials (fEPSPs) were recorded using a MultiClamp 700B (Molecular Devices, California, USA) and concomitant Clampex 10.0 software. Responses were evoked in the CA1 of the hippocampus, by stimulating the Schaffer collaterals (Figure 1) using a concentric bipolar electrode (FHC, Maine, USA) coupled to a stimulus isolator (A365, World Precision Instruments, Florida, USA) and a Master8 stimulator (A.M.P.I., Israel). Responses were measured by a chloride coated silver wire, within an ACSF filled glass electrode (20 MΩ), and positioned in the stratum radiatum of the CA1. The electrode was pulled from borosilicate glass (outer diameter = 1.5 mm and inner diameter = 0.8 mm (Garner Glass, California, USA)) on a DMZ universal puller (Zeitz Instruments, Martinsreid, Germany). 

Input/output-curves (I/O-curves) were performed by increasing the stimulation intensity by 0.05 mA every 15 seconds, starting from 0 mA and concluding at 0.75 mA. One fEPSP was recorded for each stimulation intensity during each experiment. The duration of the stimulation pulse was 0.1 ms, as was the case for all stimulating pulses for all experiments. Input/output measurements were also performed on slices before the initiation of PPF and trains, in order to find the maximum response, and thereby to set the intensity for all experiments to 50% of the maximum evoked fEPSP amplitude. Paired-pulse facilitation (PPF) was performed on slices from P30-P60 wobbler mice and age-matched littermate controls, with paired fEPSPs evoked every 15 seconds. The interstimulus intervals were (in ms): 25, 50, 75, 100, 125, 150, 200 and 300. Trains were recorded in slices prepared from P17-P21 and P45-P60 wobbler mice and age-matched littermate controls by the application of 10 or 200 pulses. A train was evoked every 15 seconds, with three consecutive sweeps for each type of train. The protocols were always evoked in the same order of ascending intensity: 10 pulses at 10 Hz; 10 pulses at 20 Hz, 10 pulses at 50 Hz, 200 pulses at 20 Hz, and 200 pulses at 50 Hz; and with a three minute pause between each type of train. 

### Field potential acquisition and analysis

**Figure 2:**
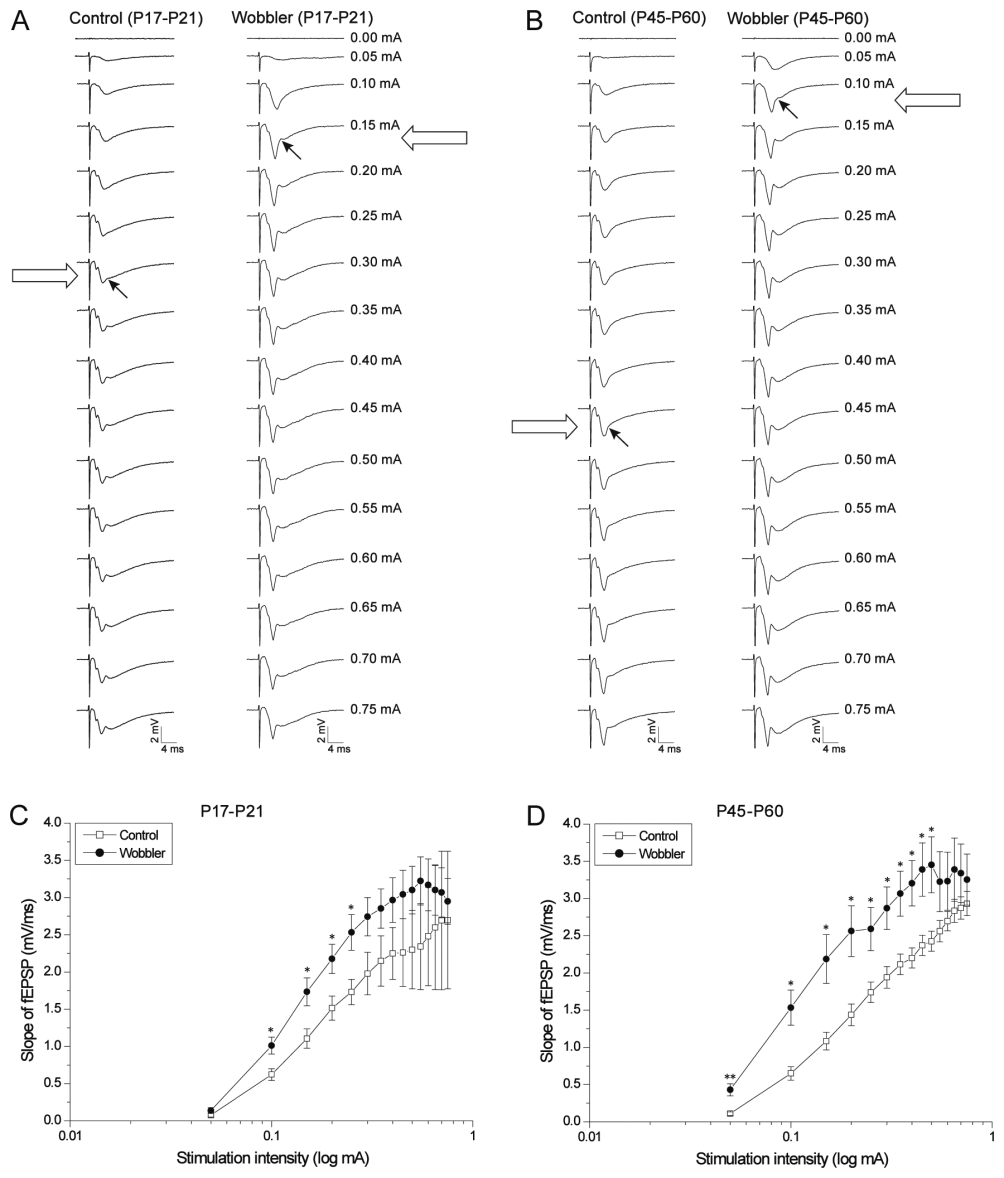
Wobbler mice exhibit increased synaptic excitation. (A-B) Representative fEPSP traces recorded during I/O-curve studies in presymptomatic (P17-P21) and symptomatic (P45-P60) wobbler mice (right sides) and controls (left sides). The arrows illustrate the first observation of a population spike in the given experiment, and it can be appreciated that the population spikes occur at lower stimulation intensities in wobbler mice. (C) The I/O-curves of both the presymptomatic (P17-P21) (left) and the symptomatic (P45-P60) (right) wobbler mice are shifted compared to the control mice, as evidence of increased synaptic excitation (P17-P21: control: n = 10 slices/5 mice, wobbler: n = 16 slices/6 mice. P45-P60: control: n = 12 slices/7 mice, wobbler: n = 16 slices/7 mice). T-test: *P<0.05; **P<0.01. Error bars represent SEM.

**Figure 3:**
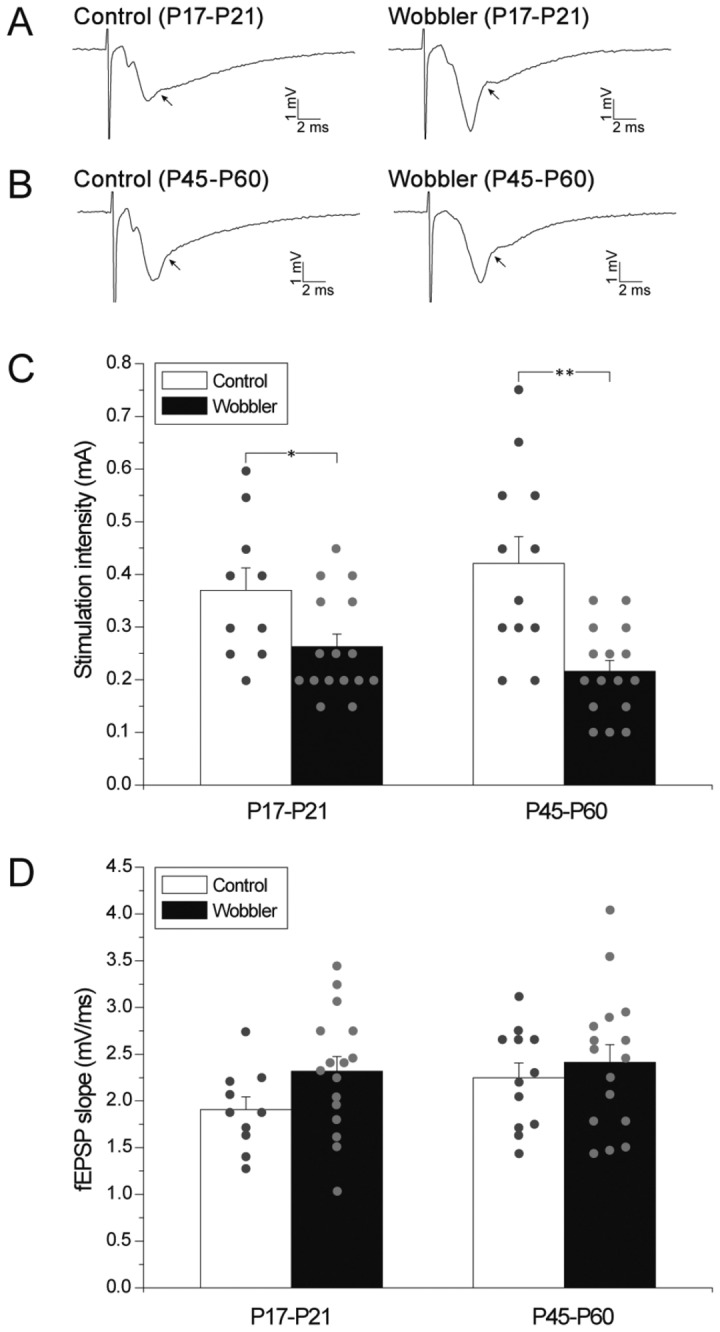
Population spikes are evoked at lower stimulation intensities, but same fEPSP size, in wobbler mice. (A) Cut-out from Figure 2A: fEPSP traces showing the first observation of a population spike, resulting from CA1 pyramidal cell firing during I/O-curve recordings in presymptomatic (P17-P21) wobbler mice (right) and controls (left). The arrows point to what was defined as the initial population spikes (corresponding to 0.15 mA and 0.3 mA, respectively, see also arrows in Fig. 2A). The development of the population spikes can be appreciated in Figure 2A. (B) Cut-out from Figure 2B showing fEPSP traces recorded during experiments in wobbler mice (right) at the symptomatic phase (P45-P60) and control mice (left), with arrows illustrating the first observed population spikes (corresponding to 0.1 mA and 0.45 mA, respectively, see also arrows in Fig. 2B). The progression of the population spikes can be seen in Figure 2B. (C) The average stimulation intensity needed to evoke a population spike in P17-P21 (left) and P45-P60 (right) wobbler mice and control are illustrated by the diagrams. The small circles demonstrate the stimulation intensity distribution. (D) The fEPSP slopes corresponding to the first observation of a population spike are similar in the four groups. Small circles illustrate the individual slope measurements. (P17-P21: control: n = 10 slices/5 mice, wobbler: n = 16 slices/6 mice. P45-P60: control: n = 12 slices/7 mice, wobbler: n = 16 slices/7 mice). T-test: *P<0.05; **P<0.01. Error bars represent SEM.

Analyses of the slopes of the recorded fEPSPs were performed in Clampfit 10.0 (Molecular Devices, California, USA). The slopes were estimated as illustrated in Figure 1 B. The first occurrence of population spikes upon increasing stimulation was judged visually as an opposing deflection in the fEPSP. Traces with the first signs of a population spike are illustrated in Figure 2 and Figure 3. The development of the population spikes can be appreciated in Figure 2A and 2B. In all experiments statistical significance was evaluated using unpaired student’s t-tests. Additionally, Kolmogorov-Smirnov (KS) tests (http://www.physics.csbsju.edu/stats/KS-test.html) were performed on the complete data sets underlying I/O-curves of wobbler mice versus controls at both P17-P21 and at P45-P60. KS-tests were performed as a second test to evaluate the complete curves, and not only the individual stimulation intensity points (as previously described [46]).

### Immunohistochemical procedures

Wobbler mice and age matched control littermates of both sexes, at either P18-P19 or at P56 were used for immunohistochemical analysis. Mice were anesthetized with Mebumal and transcardially perfused with phosphate-buffered saline (PBS), followed by 4% PFA in PBS. The brains were then postfixed for 2-4 hours at 4°C in the same solution, before being transferred to 30% sucrose in PBS, and stored at 4°C until they sank to the bottom. 40 μm coronal slices containing the hippocampal formation (approximately from Bregma: -1 to -3), were cut on a Leica CM1900 cryostat. 

Free-floating slices were washed in PBS, and in ethanol to remove endogenous peroxidase activity. After incubation with the blocking solution containing 3% normal donkey serum and 0.3% Triton X-100 in PBS, the slices were incubated with the primary antibody (PVG-214, 1:5000, SWANT) at 4°C overnight. Slices were since incubated with the secondary antibody (Biotin-SP-conjugated Affinity Pure Donkey Anti-Goat IgG, 1:2000, Jackson ImmunoResearch) at 4°C overnight, and then using a standard ABC kit (Vector Laboratories) for an hour at room temperature. Peroxidase activity was revealed by 0.02% DAB, with 0.01% H2O2. Slices were mounted on gelatinized glass slides and cover slipped with DPX mounting solution (Fluka). 

### Image acquisition and analysis

Parvalbumin positive neurons were photographed using bright field microscopy on an inverted AF6000LX microscope (Leica, Germany). The captured pictures were patched together in Adobe Photoshop, with no alterations to the picture composition, to visualize all areas of the hippocampal formation in one image. We performed a blinded study by two individuals in which the parvalbumin positive cell bodies in the soma layer of CA1 and CA2+3, and in the hilus and the granule cell layer of the dentate gyrus were counted in slices containing both CA1-3 and the dentate gyrus. The average number of parvalbumin positive neurons per slice was calculated for each area for wobbler mice and controls at P18-19 and at P56. Data were analyzed statistically using unpaired student’s t-tests to determine if changes in cell numbers were significant. 

## Results

### Input/output relationships reveal increased synaptic excitation in wobbler mice

To investigate the synaptic properties in a neuronal network, afferent fibers can be electrically stimulated and the resulting synaptic response recorded. Here we took advantage of a part of the well-established trisynaptic network of the hippocampus [47]: We stimulated the Schaffer collaterals using a graded stimulation and recorded the resulting fEPSP (Figure 1). Input/output curves were constructed by plotting the slopes of the recorded fEPSPs versus the stimulation intensities. Figure 2B illustrates the fEPSP sweeps of the I/O-curves from symptomatic (P45-P60) wobbler mice and age matched littermate controls. The I/O-curve of the symptomatic wobbler mice is shifted significantly leftward compared to the I/O-curve of the controls (Figure 2D). This shift was determined by student’s t-tests on the slopes for a given stimulation intensity found in wobbler mice and controls, and additionally by a KS-test (as previously described [46]). These tests indicate that the synaptic excitation of Schaffer collateral synapses in the hippocampus of the wobbler mouse is significantly increased during the symptomatic phase. 

In order to establish if the increased excitation is already present during the presymptomatic phase, I/O-curves on P17-P21 wobbler mice and littermate controls were also performed (Figure 2A), and similar to the symptomatic mice we found a significant leftward shift of the I/O-curve from the presymptomatic wobbler mice, by t-tests and a KS-test (Figure 2C). This shift indicates that the synaptic excitation in the hippocampus of the wobbler mouse is indeed increased already at this early stage of the disease, prior to the expression of visible phenotypic symptoms. 

### Threshold changes for pyramidal cell firing in wobbler mice

We next aimed to analyze the intrinsic excitability of the CA1 pyramidal cells. When pyramidal cells fire action potentials it can be observed as a positive deflection in the fEPSP recorded in stratum radiatum (Figure 3A and 3B). The stimulation intensity at which a population spike is first observed can be used as a measure of the excitability of a neuronal population during fEPSP recordings [48]. To measure how much afferent stimulation was needed to elicit pyramidal cell firing we found the minimal stimulation intensity required to evoke a population spike, and in symptomatic wobbler mice (P45-P60) the mean stimulation intensity necessary was significantly lower than in the littermate controls (Figure 3C; wobbler 0.22 ± 0.02 mA; control 0.42 ± 0.05 mA). Likewise, in presymptomatic wobbler mice (P17-P21) the stimulation intensity required to evoke a population spike was significantly lower than for age matched control littermates (Figure 3C; wobbler 0.26 ± 0.02 mA; control 0.37 ± 0.04 mA). This demonstrates that 1.4 to 2.0-fold less stimulation is needed to evoke APs in the pyramidal cells of the wobbler hippocampus, depending on the developmental stage. 

Since the slope of the fEPSP is a measure of the degree of postsynaptic activation of glutamate receptors [49], we next analyzed how the size of the fEPSP slope correlated to the occurrence of population spikes in the four groups of animals (Figure 3D; P17-P21: wobbler 2.32 ± 0.16 mV/ms; control 1.91 ± 0.14 mV/ms; P45-P60: wobbler 2.41 ± 0.19 mV/ms; control 2.25 ± 0.16 mV/ms). This analysis revealed that an equal strength of synaptic activation was needed to elicited action potentials in CA1 pyramidal cells in slices prepared from wobbler mice and controls; suggesting that the observed increase in excitability is primarily caused by an increase of the excitatory transmission.

### Presynaptic vesicle release probability is normal in wobbler mice

**Figure 4:**
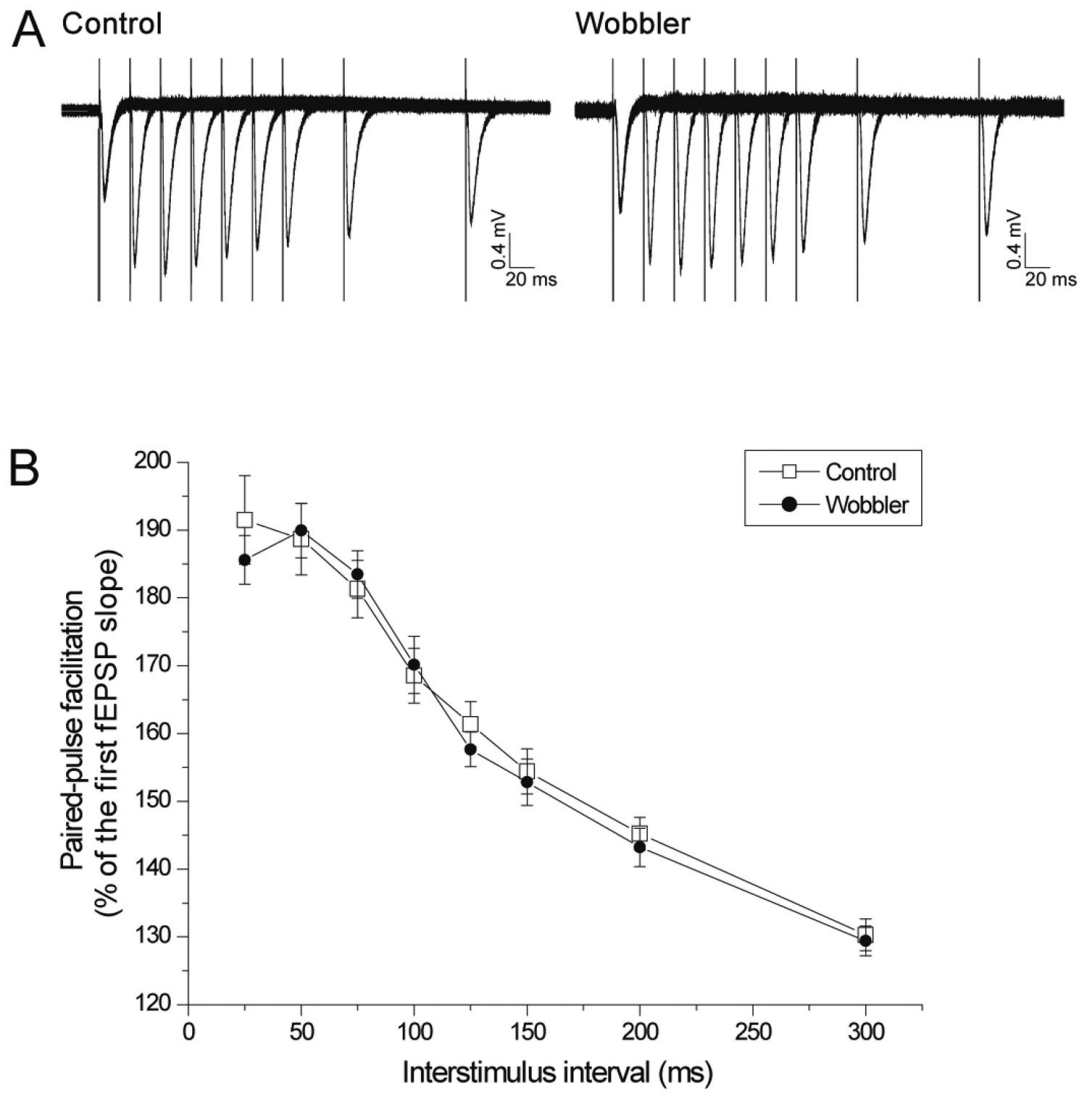
Normal release probability in wobbler mice. (A) Representative fEPSP traces recorded during paired-pulse facilitation (PPF) performed in brain slices from a wobbler mouse (left) and a control mouse (right). (B) The fEPSP slope of the second pulse was normalized to the first pulse in the respective sweep, and in each slice, the slopes of three sweeps were averaged for each stimulation interval. No significant difference was seen in the magnitude of PPF between wobbler mice and controls (wobbler: 19 slices/8 mice and control: 18 slices/9 mice). T-test: P<0.05. Error bars represent SEM.

Impaired axonal transport is believed to be a cause of ALS [50], and has specifically been shown to be negatively affected in the symptomatic wobbler mouse [51-53]. Therefore, in order to test the presynaptic functionality at the Schaffer collateral-pyramidal cell synapses in the CA1, we performed paired-pulse facilitation (PPF) experiments on symptomatic wobbler mice and control littermates (Figure 4). Two responses evoked with a brief interstimulus interval reveal information about the neurotransmitter release probability [54]. Figure 4A illustrates fEPSP sweeps recorded from a wobbler mouse and a control (P30-P60) during the experiments, and shows that the controls and the wobbler mice express similar PPF, with no difference in magnitude or time course (Figure 4B). These paired-pulse analyses indicate that the presynaptic vesicle release probability is normal in the wobbler model.

### Repetitive stimulation and synaptic depletion give normal responses in wobbler mice

**Figure 5:**
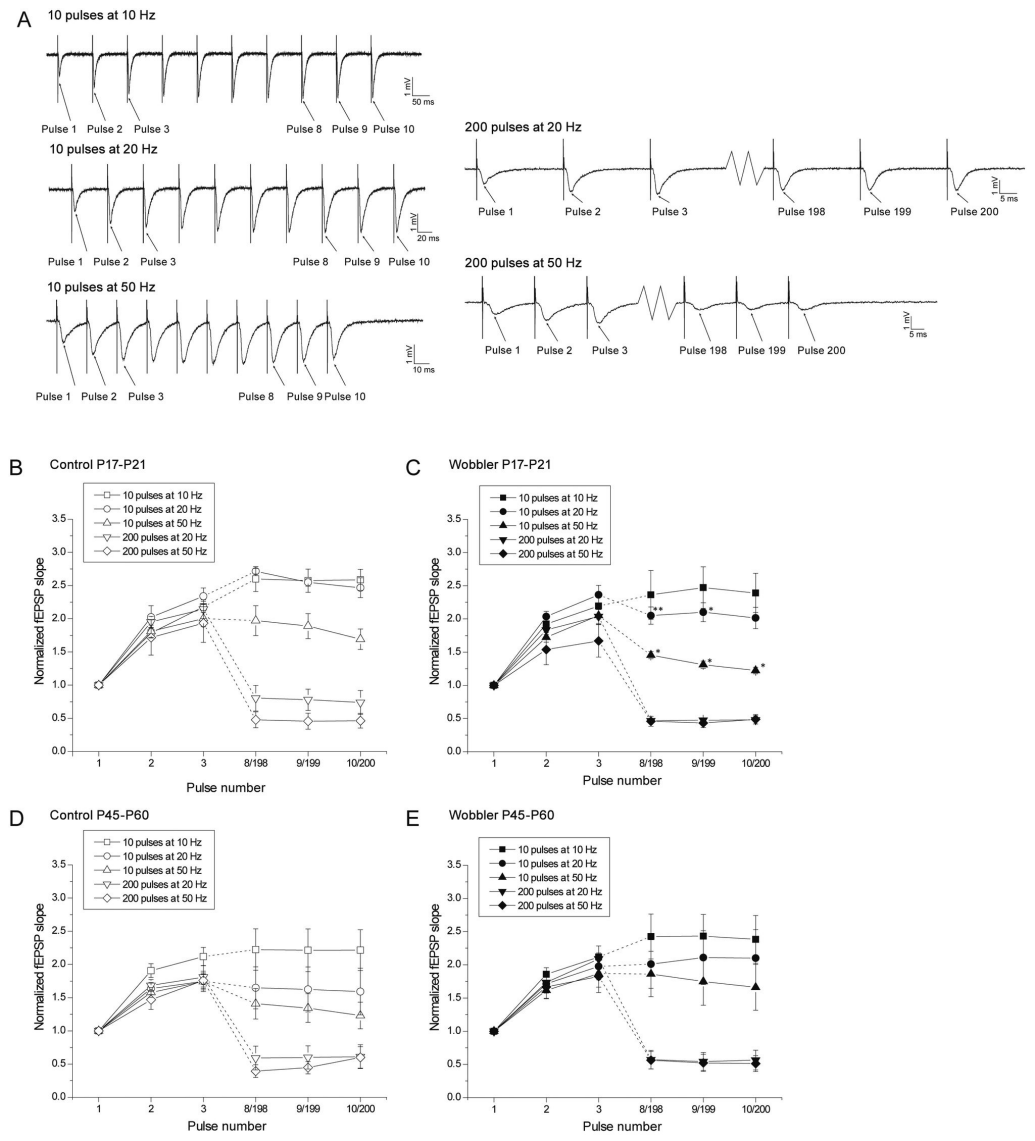
Wobbler mice do not exhibit presynaptic impairments. (A) fEPSPs recorded during train stimulation of the Schaffer collaterals, in control mice at P17-P21. Arrows illustrate the pulses employed for the analyses: the slopes of the first three and the last three pulses (pulses 1, 2, 3, 8, 9, and 10 for the types of trains consisting of 10 pulses, and pulses 1, 2, 3, 198, 199, and 200 for the types of trains consisting of 200 pulses) from each type of train were normalized to the slope of the first pulse in the given trains. (B-E) No physiologically relevant differences were observed in short-term synaptic plasticity when comparing wobbler mice and control littermates during the presymptomatic phase (comparing B to C) or the symptomatic phase (comparing D to E). However, a few results reached statistical significance when testing the trains of the same intensity in wobbler mice against controls, as indicated by * (P<0.05) or ** (P<0.01) in the figure (C compared to B) (t-test). Note the shifts in pulse number. (P17-P21: control: n = 5 slices/4 mice, wobbler: n = 6 slices/6 mice. P45-P60: control: n = 10 slices/6 mice, wobbler: n = 9 slices/7 mice). Error bars represent SEM.

**Figure 6:**
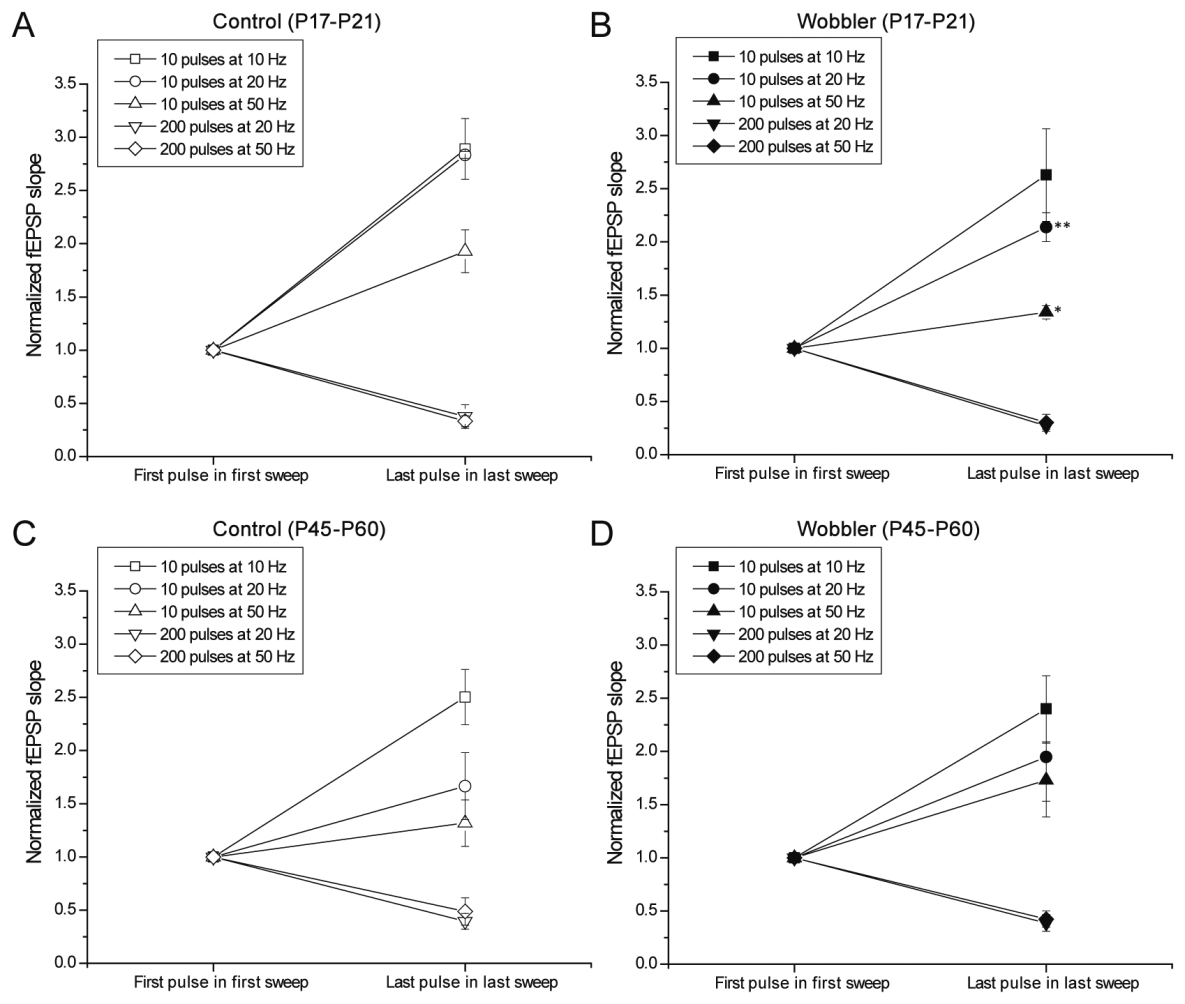
Synaptic depletion is normal in wobbler mice. Comparisons of the sizes of the very first and very last pulses evoked during the three consecutive trains for each stimulation protocol. Responses (pulses number 10 from the types of trains consisting of 10 pulses, and pulses number 200 from the types of trains consisting of 200 pulses) were normalized to the very first pulse in the first sweep of the three consecutive trains. No physiologically relevant differences were seen between the wobbler mice (B+D) and control littermates (A+C) during the pre-symptomatic phase (P17-P21) (A+B) or the symptomatic phase (P45-P60) (C+D). Two points of statistical significance was however reached in the results, indicated by * (P<0.05) and ** (P<0.01) in the Figure (B) (t-test). Error bars represent SEM.

The normal PPF does not necessarily preclude presynaptic mechanisms from playing a role in the wobbler phenotype. It is possible that stronger and longer lasting stimulation could show alterations which were not revealed by the PPF stimulation. Accordingly we performed train stimulation (Figure 5A), which like PPF primarily tests presynaptic functionality, but constitutes longer and stronger requirements of vesicle release [55]. Trains of 10 pulses were applied at 10, 20 and 50 Hz, as well as longer trains of 200 pulses at 20 and 50 Hz. The train experiments were analyzed in three different ways (Figures 5,6 and Figures S1-S4). 

Initially the responses from the different types of trains were analyzed by normalizing the first three and the last three pulses of the first sweeps to the first pulse in the respective sweep (Figure 5). Overall wobbler mice and controls responded comparably in regard to size development through the various trains, both in the presymptomatic and symptomatic phase. During analyses we did nevertheless find some points of statistical significant differences (Figure 5C), but we argue that these are not of physiological relevance since they occur only in presymptomatic wobblers and only at the short trains of stimuli. The second analysis was of the development of the slopes of the responses during the three consecutive trains in the four groups (Figures S1-S4), and no physiological relevant differences were found here either, even though a few points of statistical significance was observed (Figures S2 and S4). 

In the third analysis, a synaptic depletion study, the effect of a very strong repetitive stimulation was tested by normalizing the size of the very last response to the very first pulse in the given type of train (Figure 6). If the wobbler synapses displayed any impairment in the ability to release glutamatergic vesicles upon repetitive stimulation it would have been revealed by this final synaptic depletion analysis. However, since no physiological relevant differences were found between controls and wobbler mice of the presymptomatic phase (P17-P21) or of the symptomatic phase (P45-P60) in this, or the two other analyses, the wobbler mouse does not demonstrate a reduced capacity for release of glutamatergic vesicles at Schaffer collateral synapses, even upon high rate stimulation. 

### Symptomatic wobbler mice demonstrate decreased numbers of parvalbumin positive interneurons

**Figure 7:**
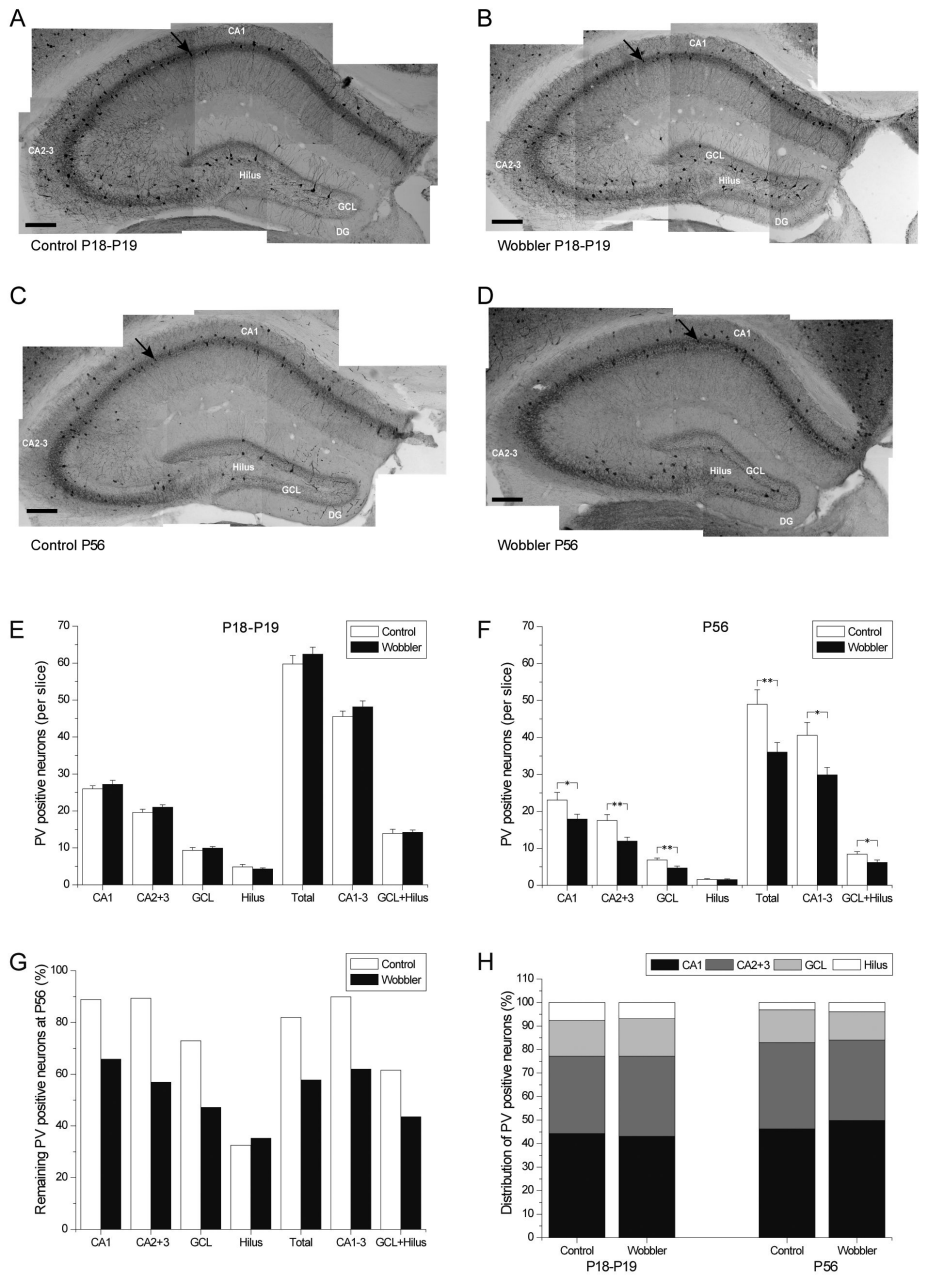
Reductions in parvalbumin (PV) positive interneurons in the hippocampus of the wobbler mice. (A-D) Parvalbumin (PV) positive interneurons (illustrated by arrows) were immunohistochemically stained and counted in slices of the hippocampal formation of presymptomatic (P18-P19) wobbler mice (B) and controls (A). Symptomatic (P56) wobbler mice (D) and controls (C) were also stained. Scale bars represent 200 µm. (E) During the presymptomatic phase (P18-P19) no significant differences were found in the number of parvalbumin positive interneurons per slice in any area of the hippocampal formation between wobbler and control mice. (F) At the symptomatic phase (P56) a reduction was found in all examined areas, except in the hilus, in the wobbler mice. (G) A reduction in parvalbumin positive interneurons is seen in both wobbler mice and control mice between P18-P19 and P56; however the reduction in the wobbler mice was greater in most areas. (H) The distribution of the parvalbumin positive interneurons at the different areas are similar in wobblers and controls at the presymptomatic phase (P18-P19), and remain similar at the symptomatic phase (P56), t-test: P>0.05. (P18-19: control: n = 46 slices/4 mice, wobbler: n = 48 slices/4 mice. P56: control: n = 34 slices/4 mice, wobbler: n = 31 slices/3 mice). T-test: *P<0.05; **P<0.01. Error bars represent SEM.

A general decrease in the number of GABAergic interneurons in the hippocampus of the wobbler mouse was recently reported [30], and the number of neocortical parvalbumin positive interneurons is reduced in wobbler mice [24]. Consequently, we executed immunohistochemical staining against parvalbumin in the hippocampal formation (Figures 7A-D). We aimed to investigate if the putative connection between the reduced number of parvalbumin positive inhibitory neurons of the cortex and increased cortical excitability in both ALS patients [8,10,56] and in wobbler mice [24] might also be present in the hippocampus of wobbler mice. 

In the presymptomatic mice (P18-P19) no significant difference in the number of parvalbumin positive interneurons per slice was observed, in any of the investigated areas of the hippocampal formation (**CA1**: wobbler 27.19 ± 1.07 cells/slice, control 26.00 ± 0.83 cells/slice; **total**: wobbler 62.41 ± 1.90 cells/slice, control 59.75 ± 2.29 cells/slice) in wobbler mice as compared to control mice (Figure 7E and Table S1). However in symptomatic wobbler mice (P56) the number of parvalbumin positive interneurons was significantly reduced in all areas of the hippocampal formation (**CA1**: wobbler 17.90 ± 0.29 cells, control 23.07 ± 2.05 cells/slice; **total**: wobbler 36.05 ± 2.57 cells/slice, control 48.99 ± 3.95 cells/slice), except the hilus, compared to littermate control mice (Figure 7F and Table S1). 

Reductions in the average number of parvalbumin positive interneurons were observed in all areas of the hippocampal formation in both controls and wobbler mice between P18-P19 and P56 (Figure 7G). However, the reductions were considerably more extensive in the wobbler mice (total reduction: wobbler 42%; control 18%), thereby giving rise to the significantly reduced number of parvalbumin positive neurons between controls and wobbler mice at P56. As determined by t-tests on the number of parvalbumin positive interneurons in the different hippocampal areas, the distribution of the parvalbumin positive cells was similar in wobbler mice and controls at P18-P19 (Figure 7H). Even though a larger reduction in the number of these cells was observed in the wobbler mouse than in the controls, the distribution of the cells remained similar in wobbler mice and controls at P56 (Figure 7H).

## Discussion

This study demonstrates that the wobbler mouse model of ALS exhibits increased synaptic excitation with a concomitant reduction in inhibitory parvalbumin positive interneurons, which together suggest hippocampal hyperexcitability. Presynaptic indicators of glutamate vesicle release and turnover on the other hand appeared unaffected. 

### Wobbler mice display an increased synaptic excitation

We performed input/output studies in the hippocampus of wobbler mice and control littermates both from the presymptomatic and the symptomatic group. Interestingly we observed a significant leftward shift of the I/O curves obtained in wobbler mice when compared to I/O curves in the age matched control mice documenting an increased synaptic excitation in wobbler animals (Figure 2). The I/O-curves show that the hyperexcitability of the wobbler mouse precedes the onset of clinical symptoms, since increased synaptic excitation is already present during the presymptomatic phase (Figure 2). These findings correspond to studies of cortical hyperexcitability in both ALS patients and in the wobbler mouse, as ALS patients have been shown to demonstrate cortical hyperexcitability, before the onset of clinical symptoms [6,7], and wobbler mice also demonstrate neocortical hyperexcitability in the presymptomatic to early symptomatic phase [24]. Interestingly, hyperexcitability can also be observed in the SOD1(G93A) mouse model of ALS [25-28]. In accordance with the expectations, the increased excitation was also documented in the wobbler mice during the symptomatic phase of the disorder (Figure 2), suggesting that the hyperexcitability of the wobbler hippocampus is established early in the disease, and persist through the degeneration of the motor system as the symptoms of the disease progress. 

In the literature, hyperexcitability has been suggested to be the result of high levels of extracellular glutamate, which has been found in some ALS patients [57,58], or as a degeneration of the inhibitory circuits [8] including reduced levels of GABA [10]. In the presymptomatic mice, we found no difference in the number of parvalbumin positive interneurons in any area of the hippocampal formation in wobbler mice compared to control mice. This suggests that the hyperexcitability of the wobbler mouse is not only caused by degeneration of the inhibitory circuits, and would be in accordance with results from ALS patients showing that hyperexcitability is caused by a combination of excitotoxicity and reduced inhibition [8]. Various studies have tried to assess the role of glutamate in the wobbler mouse, as reviewed in Moser et al. [13]. However different areas have been examined: spinal cord [59,60], plasma [61], brain [60] or cultured astrocytes [45,62], and with divergent results. Taken together, the above mentioned studies, in addition to studies of glutamine synthetase [63,64] and glutamate transporters [45,61] are indicative that the glutamate homeostasis might be altered in the wobbler mouse [62]. Furthermore, altered AMPA receptor trafficking has been reported in another ALS model, the *ALS2* knock-out mouse [20]. By analyzing evoked AMPA receptor mediated currents in cortical neurons the authors proved that the AMPA receptor subunit composition was changed in the ALS2 knock-outs resulting in an increased number of GluR2-lacking receptors. This change caused the receptors to be more calcium permeable and therefore the neurons more susceptible to glutamate receptor-mediated neurotoxicity [20]. An altered number or composition of postsynaptic AMPA receptors, probably in combination with increased extracellular glutamate, would explain the leftward-shifted input/output relationship we observed in the wobbler mouse. 

To test for changes in the intrinsic excitability of the CA1 pyramidal neurons we studied the population spike occurrence and found that the CA1 pyramidal cells fire action potentials at similar fEPSP slopes in all groups (Figure 3D), meaning that the action potential threshold is the same in wobbler mice and controls. However, due to increased synaptic excitation in the wobbler animals, the threshold fEPSP is reached at significantly lower stimulation intensities (Figure 3C). Future studies employing patch-clamp analysis of hippocampal neurons may shed more light on these mechanisms.

### The wobbler mouse does not display presynaptic defects

Impaired axonal transport has been implicated as one of the underlying causative mechanisms both in ALS patients [50], and in the symptomatic wobbler mouse [51-53]. Anterograde axonal transport is important for the transport of cellular components such as proteins and mitochondria into the axon terminal [50]. Both retrograde and anterograde axonal transport have been shown to be impaired in the wobbler mouse [51,53], and the GARP-complex is important for the retrograde transport of endosomal derived compartments to the trans-Golgi network [65]. 

In some ALS cases, proteins like VAPB, alsin, and Figure 4, all of which are involved in endosomal trafficking, have substantiated that endosomal trafficking impairments can lead to motor neuron disease [66]. These findings suggested to us that the ability to release neurotransmitter vesicles, especially upon repetitive stimulation, might be reduced. Interestingly the wobbler mice did not show any differences in release probability during the symptomatic phase compared to the control littermates (Figure 4). Additionally, no physiologically significant differences were seen during the train experiments, regardless of whether differences in a single sweep train or consecutive sweeps of trains were considered (Figures 5,6 and Figures S1-S4). A few statistically significant differences were found between wobbler mice and controls in the train experiments, however these are most likely not physiologically relevant, as the significant differences were few and occurred inconsistently. 

Taken together the lack of differences in both the PPF experiments and in the repetitive stimulation analyses and the synaptic depletion analyses performed in both the presymptomatic and the symptomatic phases, strongly indicate that defects in presynaptic mechanisms are not involved in the phenotype of the wobbler mouse. In addition, these results show that the axonal transport defects of the wobbler mouse [51-53] does not affect the presynaptic vesicle release, at least not through changes in the release probability or the amount of neurotransmitter released from the readily releasable pool. Therefore, the studies of PPF and trains, which primarily reflect presynaptic mechanisms and the release of transmitter, strongly indicate that the presynaptic function of glutamatergic synapses is normal in the wobbler mouse. 

### Parvalbumin positive interneurons are reduced in symptomatic wobbler mice

In the young animals we observed a similar number of parvalbumin positive interneurons when comparing wobblers and controls (Figure 7E). It is however possible that the inhibitory circuits [8], and thereby the parvalbumin positive interneurons, are already partly degenerated in the wobbler mouse at the presymptomatic phase, but that the immunohistochemical staining performed here was not able to reveal that. Other types of interneurons, which are parvalbumin negative, are part of the hippocampal inhibitory circuits [31,32], and these would not be visible in this study, but they could conceivably be degenerated and therefore contribute to increased excitability. Another possibility could be that the parvalbumin positive interneurons are in fact already degenerating during the presymptomatic phase, but since only somas could be counted in this study, any degeneration of the axons or dendritic trees would not be visible. Indeed, earlier findings supporting such mechanism have been documented as CA1 interneurons displaying structural abnormalities in their dendrites, with no change in the number of somata, can be seen upon transient cerebral ischemia [67]. Defective differentiation and/or impaired synaptogenesis during development [33] is another possible explanation for inhibitory defects.

In the symptomatic phase, a significantly reduced number of parvalbumin positive interneurons were found in all investigated areas of the hippocampal formation, except the hilus, compared to control mice (Figure 7F). The cell number in the hilus was rather low at both times, and in both control and wobbler mice. The low cell number could perhaps underlie the lack of a significant difference in this area. The reduction strongly indicates that there is a decrease in GABAergic inhibition, and that it could contribute to hyperexcitability. It also lends credence to the hypothesis that the dendritic trees and axons of the interneurons could be corrupted during the presymptomatic phase. The inhibitory interneurons modulate glutamatergic transmission by feed-forward and feedback inhibition [68]. Especially, the parvalbumin positive fast-spiking basket cells mediate feedback inhibition onto the pyramidal cells and exert a strong perisomatic inhibitory control, defining the rhythm of network oscillations [69].

In both wobbler mice and control mice a regression in the number of parvalbumin positive interneurons was seen in all investigated areas of the hippocampal formation (Figure 7G), suggesting that part of the reduction in the number of interneurons can be attributed to normal post-natal pruning, which happens extensively in neuron populations, including GABAergic interneurons, during development [70,71]. However, the number of interneurons eliminated in wobbler mice is much larger than in control mice. It could be speculated that the normal pruning of interneurons is increased in the wobbler mouse, but further studies are required to investigate this. However it is possible that the hypothesized decrease in the dendritic trees and axons, caused by degeneration, would result in a decreased number of synaptic connections to other cells, and that this could lead to death of the interneurons. It is also conceivable that impairment of vesicular traffic caused by the reduced level of GARP-complex [17,18,72] could lead to a reduction in presentation of survival signals, and that the cells would therefore be removed. Whatever the mechanism, the similar proportional distribution of the remaining parvalbumin positive interneurons throughout the hippocampal formation in wobbler mice and control mice, at both the presymptomatic and the symptomatic phase (Figure 7H), could be indicative of similar mechanisms underlying the reductions in interneurons, but pointing to dysregulations in the wobbler mice.

## Conclusion

In conclusion, this study demonstrates that both presymptomatic and symptomatic wobbler mice exhibit increased synaptic excitation at Schaffer collateral synapses, suggesting hippocampal hyperexcitability. Furthermore we suggest that hyperexcitability could be caused by a concomitant reduced GABAergic inhibition due to a decreased number of parvalbumin positive interneurons. The reduced number of cell bodies was only observed in the symptomatic wobbler mice, which led us to speculate that presymptomatic mice harbor changes in their parvalbumin positive interneurons at the subcellular level. On the other hand, the wobbler mouse model of ALS does not exhibit apparent presynaptic impairments, which were analyzed by trains of high frequency stimulation and synaptic depletion analyses of the Schaffer collaterals. In summary, we have revealed more details of the early pathophysiology in asymptomatic animals. Importantly, the neuronal alterations of the wobbler mouse are not confined to the motor cortex, but can be extended to the hippocampus with electrophysiological analyses allowing us to observe hyperexcitability already in the presymptomatic mice. 

## Supporting Information

Figure S1 **The development of the size of the pulses when comparing the three consecutive trains in control mice at P17-P21.** For this figure, and Figures S2-S4, pulses were normalized to the respective pulse of the same number in the first sweep for each stimulation protocol. No difference was observed between the wobbler mice and controls in the presymptomatic phase (compare to Fig. S2). However three points of statistical significance were found between wobbler mice and controls. These were found in pulses numbers 1 and 3 of the third sweep in the trains of 200 pulses at 20 Hz, and in pulse number 2 of the third sweep in the trains of 200 pulses at 50 Hz (*P<0.05). Error bars represent SEM.
(TIF)

Figure S2 **The development of the size of the pulses when comparing the three consecutive trains in wobbler mice at P17-P21.** No difference was observed when compared to the control mice at the same age (see Figure S1). Error bars represent SEM.
(TIF)

Figure S3 **Comparison of the development of the size of the pulses during the three consecutive trains in control mice at (P45-P60).** No difference was found between the wobbler mice and controls in the symptomatic phase (compare to Figure S4). However a single point of statistical significance was found between wobbler mice and controls in pulse number 198 of the second sweep, in the trains of 200 pulses at 50 Hz (*P<0.05). Error bars represent SEM.
(TIF)

Figure S4 **Comparison of the development of the size of the pulses during the three consecutive trains in wobbler mice at P45-P60.** When compared to the control mice at the same age no difference was found (see Figure S3). Error bars represent SEM.
(TIF)

Table S1 **Number of parvalbumin positive interneurons in the various hippocampal areas (number/slice).** The table display the exact numbers from the immunohistochemical staining displayed in Figure 7. (P18-19: control: n = 46 slices/4 mice, wobbler: n = 48 slices/4 mice. P56: control: n = 34 slices/4 mice, wobbler: n = 31 slices/3 mice). ± represent SEM.
(DOCX)
