# Correction: The Wobbler Mouse Model of Amyotrophic Lateral Sclerosis (ALS) Displays Hippocampal Hyperexcitability, and Reduced Number of Interneurons, but No Presynaptic Vesicle Release Impairments

**DOI:** 10.1371/journal.pone.0121772

**Published:** 2015-03-25

**Authors:** The PLOS ONE Staff

There are several errors in the text of the article. The journal apologizes for these errors that occurred while the manuscript was prepared for publication.

There is an error in the last sentence of the second paragraph of the Introduction. “Figure 4” should read “*FIG4*.” The correct sentence is: There is also growing evidence for this connection in ALS patients, as seen by the involvement of other endosomal trafficking factors, namely VAPB [19], alsin [20,21], and *FIG4* [22] in ALS [23].

There are errors in the last two sentences of the “Genotyping” section of the Materials and Methods. The correct sentences are: For the wild type allele the primers were as follows: *Vps54—f413*; 5’-GCT TCT CTG TTG AAG CCA CA-3’ and *Vps54—wt—rev*; 5’-CCC AGA TCT CGG CCA TAT TTA-3’ resulting in a band at about 415 base pairs. The wobbler allele was identified by the primer pair: *Vps54—wr—f*; 5’-AGG CCT TAA AGA TCT GGA TCA-3’ and *Vps54 rev255*; 5’-TGC TCC TTA CTC AGG GAT GC-3’ giving a band at about 260 base pairs. Annealing temperature was 63°C.

There are errors in the second sentence of the “Preparation of brain slices for electrophysiology” section of the Materials and Methods. The correct sentence is: The brains were carefully dissected out and immediately transferred to ice-cold artificial cerebrospinal fluid (ACSF, in mM): 126 NaCl, 2.5 KCl, 1.25 NaH_2_PO_4_, 2.5 CaCl_2_, 1.3 MgCl_2_, 26 NaHCO_3_, 10 D-glucose, bubbled with carbogen (5% CO_2_–95% O_2_).

There is an error in the penultimate sentence of the “Immunohistochemical procedures” section of the Materials and Methods. The correct sentence is: Peroxidase activity was revealed by 0.02% DAB, with 0.01% H_2_O_2_.
