# Supplementary figures and images for: The Wobbler Mouse Model of Amyotrophic Lateral Sclerosis (ALS) Displays Hippocampal Hyperexcitability, and Reduced Number of Interneurons, but No Presynaptic Vesicle Release Impairments
Source: PLoS One. 2013 Dec 11;8(12):e82767. doi: 10.1371/journal.pone.0082767 (PMC3859636; doi:10.1371/journal.pone.0082767)

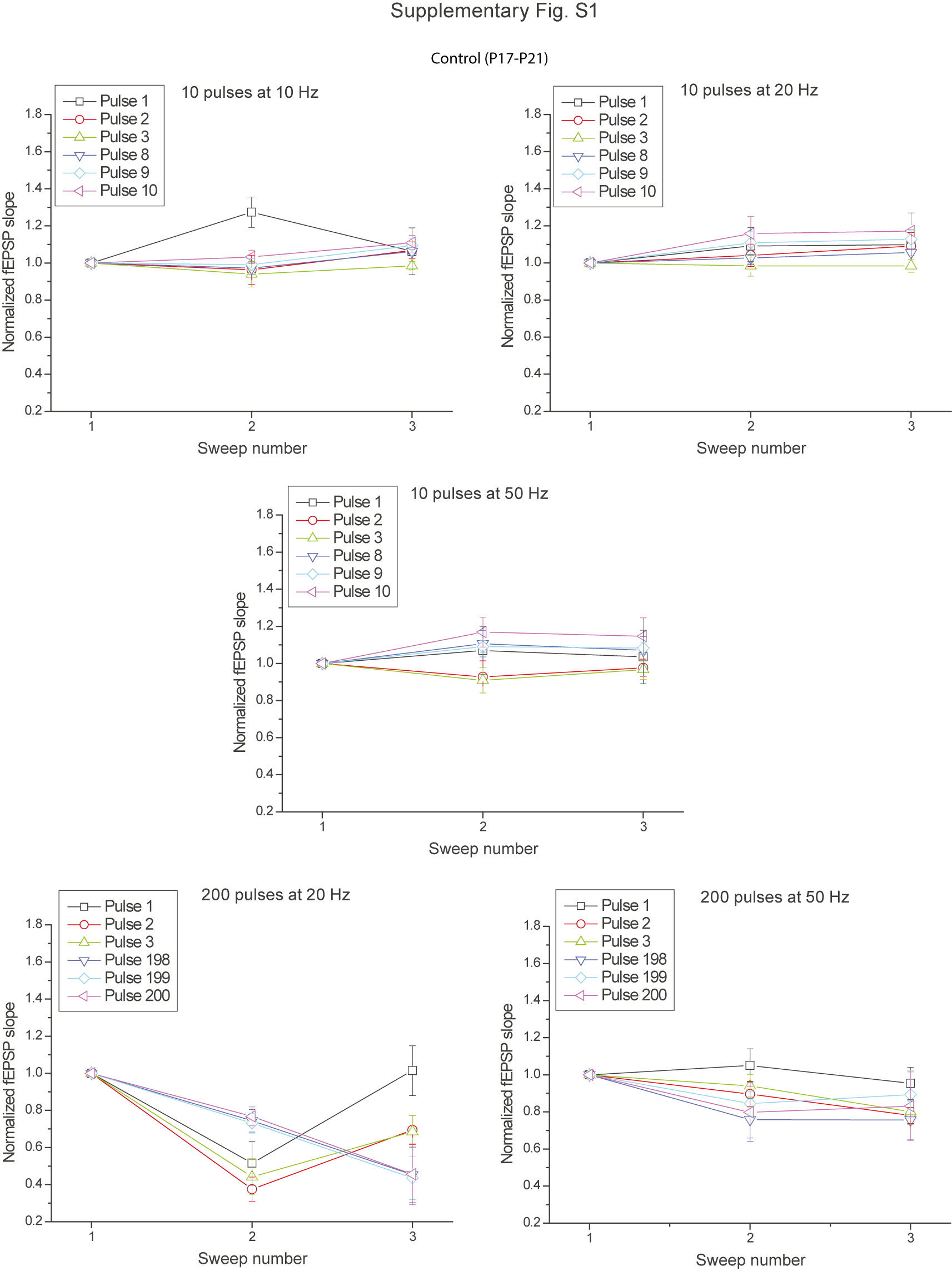

Supplement: Figure S1 — The development of the size of the pulses when comparing the three consecutive trains in control mice at P17-P21. For this figure, and Figures S2-S4, pulses were normalized to the respective pulse of the same number in the first sweep for each stimulation protocol. No difference was observed between the wobbler mice and controls in the presymptomatic phase (compare to Fig. S2). However three points of statistical significance were found between wobbler mice and controls. These were found in pulses numbers 1 and 3 of the third sweep in the trains of 200 pulses at 20 Hz, and in pulse number 2 of the third sweep in the trains of 200 pulses at 50 Hz (*P<0.05). Error bars represent SEM. (TIF) [file pone.0082767.s001.tif]

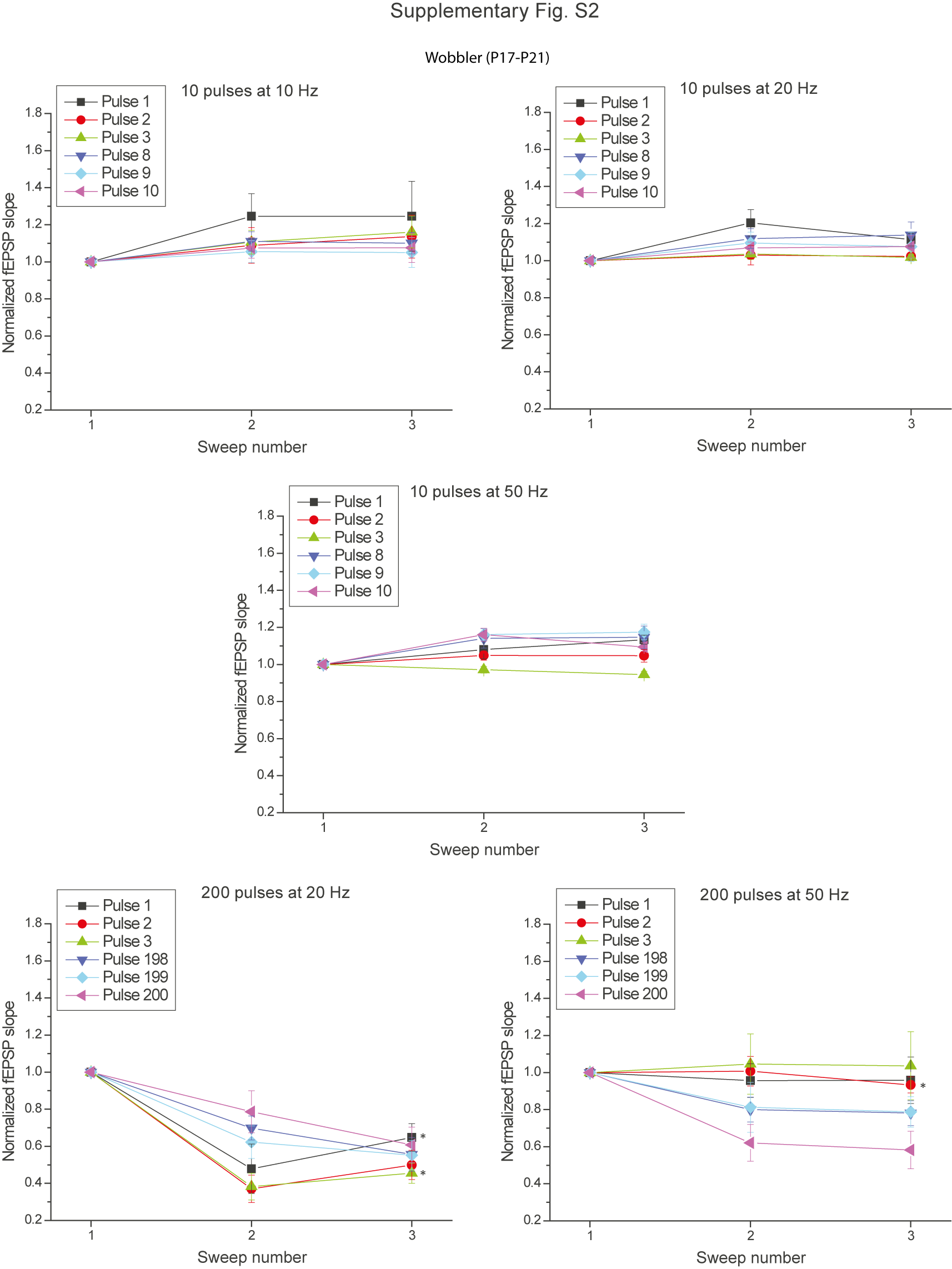

Supplement: Figure S2 — The development of the size of the pulses when comparing the three consecutive trains in wobbler mice at P17-P21. No difference was observed when compared to the control mice at the same age (see Figure S1). Error bars represent SEM. (TIF) [file pone.0082767.s002.tif]

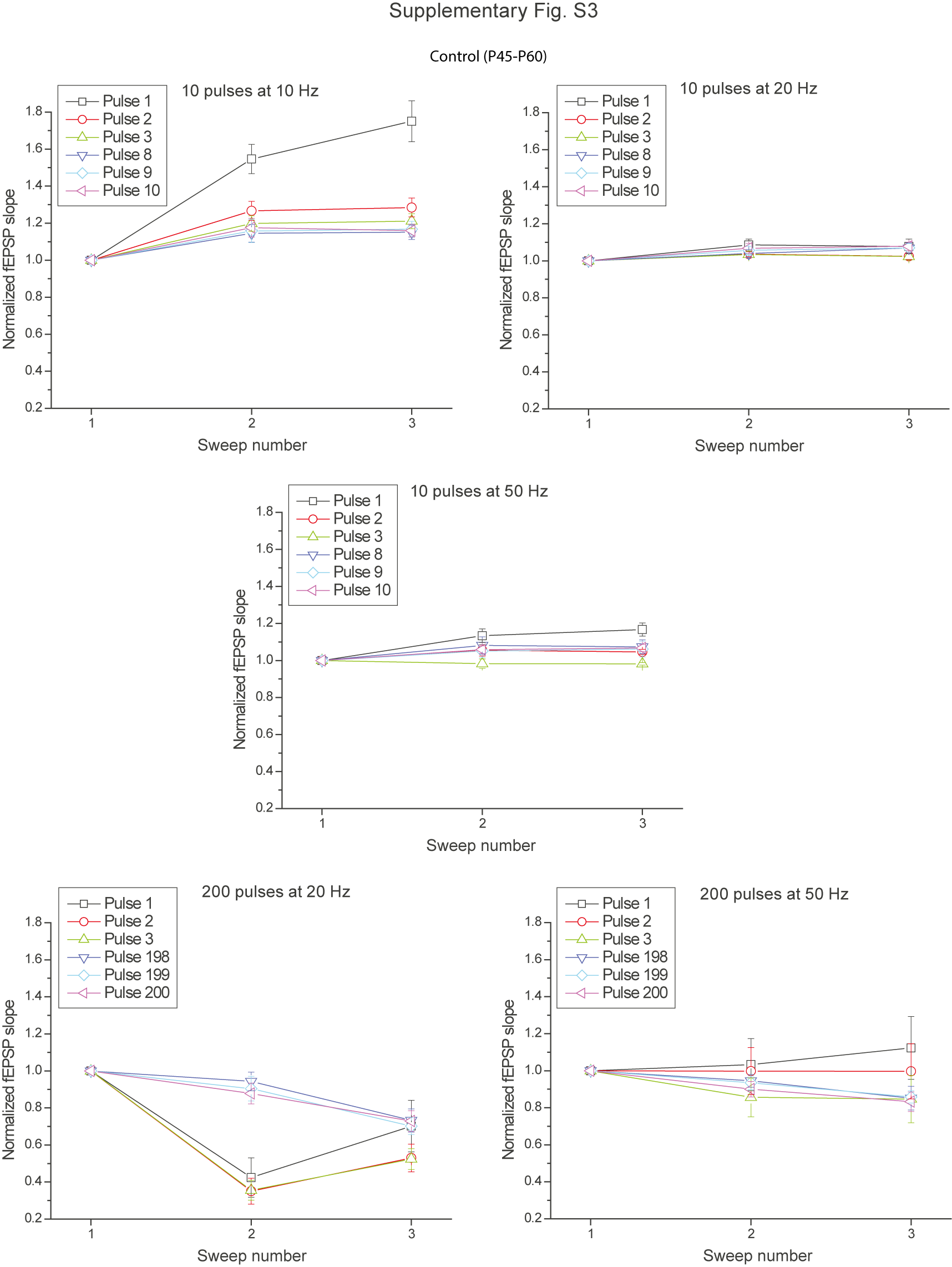

Supplement: Figure S3 — Comparison of the development of the size of the pulses during the three consecutive trains in control mice at (P45-P60). No difference was found between the wobbler mice and controls in the symptomatic phase (compare to Figure S4). However a single point of statistical significance was found between wobbler mice and controls in pulse number 198 of the second sweep, in the trains of 200 pulses at 50 Hz (*P<0.05). Error bars represent SEM. (TIF) [file pone.0082767.s003.tif]

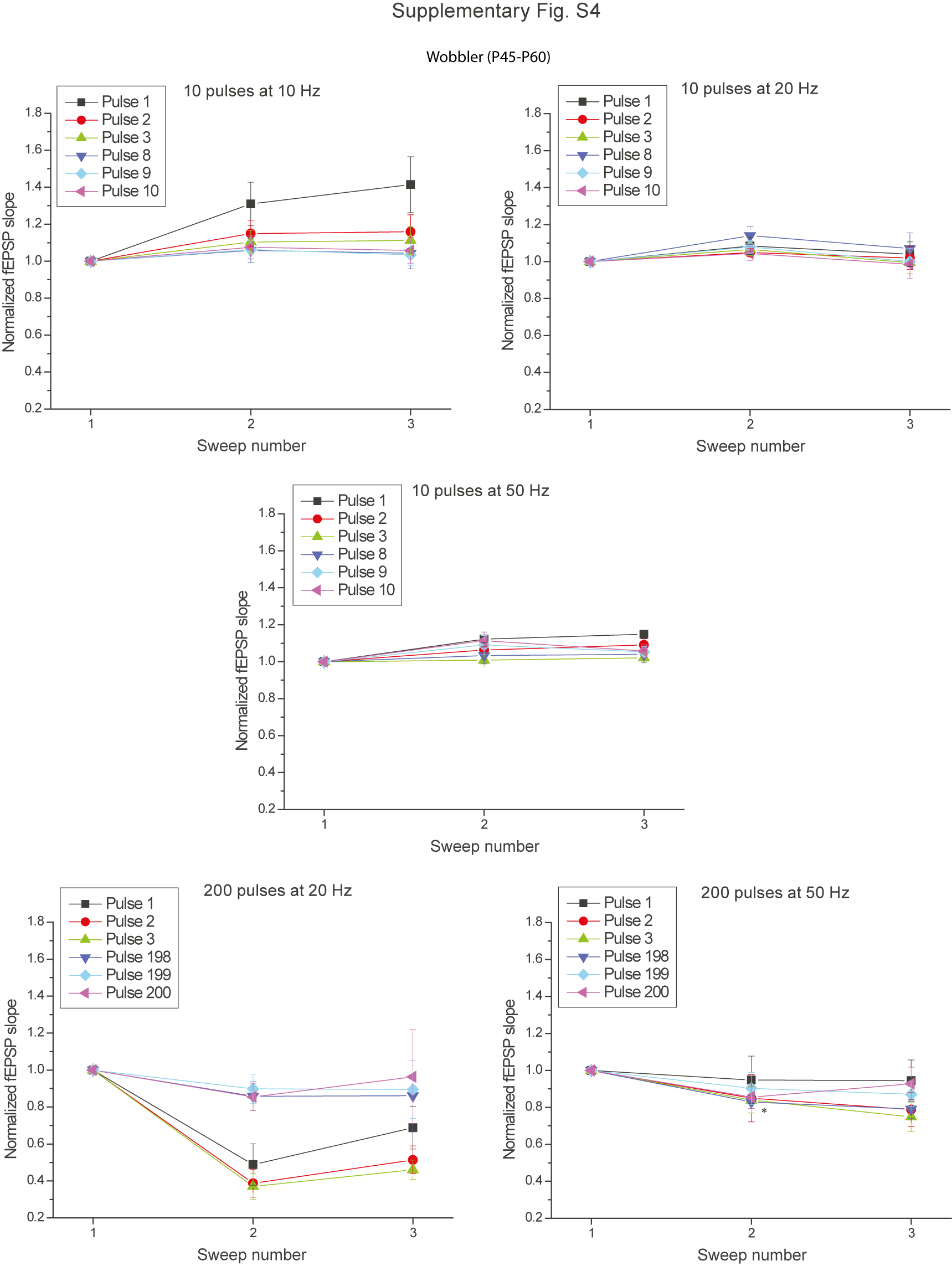

Supplement: Figure S4 — Comparison of the development of the size of the pulses during the three consecutive trains in wobbler mice at P45-P60. When compared to the control mice at the same age no difference was found (see Figure S3). Error bars represent SEM. (TIF) [file pone.0082767.s004.tif]
