# Supplementary material for: The Wobbler Mouse Model of Amyotrophic Lateral Sclerosis (ALS) Displays Hippocampal Hyperexcitability, and Reduced Number of Interneurons, but No Presynaptic Vesicle Release Impairments
Source: PLoS One. 2013 Dec 11;8(12):e82767. doi: 10.1371/journal.pone.0082767 (PMC3859636; doi:10.1371/journal.pone.0082767)
Supplement: Table S1 — Number of parvalbumin positive interneurons in the various hippocampal areas (number/slice). The table display the exact numbers from the immunohistochemical staining displayed in Figure 7. (P18-19: control: n = 46 slices/4 mice, wobbler: n = 48 slices/4 mice. P56: control: n = 34 slices/4 mice, wobbler: n = 31 slices/3 mice). ± represent SEM. (DOCX) [file pone.0082767.s005.docx]

Table S1

|  | CA1 | CA2-3 | GCL | Hilus | Total | CA1-3 | GCL+Hilus/  DG |
| --- | --- | --- | --- | --- | --- | --- | --- |
| Wobbler  P18-P19 | 27.19 ±  1.07 | 21.00 ±  0.67 | 9.92 ±  0.43 | 4.30 ±  0.27 | 62.41 ±  1.90 | 48.19 ±  1.59 | 14.22 ±  0.61 |
| Control  P18-P19 | 26.00 ±  0.83 | 20.00 ±  0.82 | 9.36 ±  0.70 | 4.84 ±  0.70 | 59.75 ±  2.29 | 45.55 ±  1.44 | 13.64 ±  0.89 |
| Wobbler  P56 | 17.90 ± 1.29 | 11.95 ± 1.04 | 4.68 ±  0.53 | 1.52 ±  0.21 | 36.05 ± 2.57 | 29.85 ± 2.07 | 6.19 ±  0.67 |
| Control  P56 | 23.07 ± 2.05 | 17.51 ± 1.61 | 6.82 ±  0.55 | 1.57 ±  0.24 | 48.99 ± 3.95 | 40.59 ±  3.50 | 8.40 ±  0.69 |

**Table S1. Number of parvalbumin positive interneurons in the various hippocampal areas (number/slice).** (P18-19: control: n = 46 slices/4 mice, wobbler: n = 48 slices/4 mice. P56: control: n = 34 slices/4 mice, wobbler: n = 31 slices/3 mice). ± represent SEM.
